# Complete genome sequence of *Leclercia adecarboxylata* strain TB492 isolated from flue-cured tobacco leaves

**DOI:** 10.1128/mra.01069-25

**Published:** 2026-03-18

**Authors:** Chen Liu, Zhen Wang, Jiandong Zhang, Lu Han, Jinbin Wei, Kai Song, Hongjing Yang, Zhipeng Zang, Yuzhen Gao, Liang Wang, Tao Jiang, Subo Ran

**Affiliations:** 1 Gansu Tobacco Industry Co., Ltd., Lanzhou, China; 2 Enzyme Research and Development Department, Hzymes Biotechnology Co., Ltd., Wuhan, China; University of Strathclyde, Glasgow, United Kingdom

**Keywords:** flue-cured tobacco leaves, *Leclercia adecarboxylata*, complete genome sequence

## Abstract

Here, we announce the complete genome sequence of *Leclercia adecarboxylata* strain TB492, isolated from flue-cured tobacco leaves. The genome is composed of one closed contig with a length of 4,734,680 bp, resulting in 100.0× coverage containing 4,493 genes, with a GC content of 56.5%.

## ANNOUNCEMENT

Various functional microorganisms can be isolated from flue-cured tobacco leaves (1, 2). To isolate carotenoid-degrading strains, 5 g of chopped flue-cured tobacco samples (harvested from Yunnan Province, China, and aged for 1 year) were washed with sterile water using an oscillating motion. The suspension was filtered through sterile gauze. The filtrate was concentrated by centrifugation at 4°C, 10,000 rpm for 10 min. The precipitate was serially 10-fold diluted (from 10^−1^ to 10^−6^) in sterile water and cultured on plates of a selective medium with β-carotene as the sole carbon source (β-carotene 5 g/L, Na_2_HPO_4_ 6.15 g/L, KH_2_PO_4_ 1.52 g/L, (NH_4_)_2_SO_4_ 1.5 g/L, MgSO_4_·7H_2_O 0.2 g/L, CaCl_2_ 0.05 g/L, and agar 15 g/L) at 30°C for 48 h. Single colonies were isolated and repeatedly streaked for purification. A single, round, smooth colony with a clearly visible transparent zone was isolated, named TB492.

After the pure colony was cultured aerobically in 150 mL of LB medium at 30°C with shaking at 200 rpm for 6 h, genomic DNA was extracted using the Wizard Genomic DNA Purification Kit (Promega) according to the manufacturer’s protocol and quantified using a TBS-380 fluorometer (Turner BioSystems Inc., Sunnyvale, CA). The same genomic DNA extraction was used for both PacBio and Illumina library preparation. For Illumina sequencing, the DNA was sheared into 400–500 bp fragments using a Covaris M220 Focused Acoustic Shearer, following the manufacturer’s protocol. Illumina sequencing libraries were prepared from the sheared fragments using the NEXTflex Rapid DNA-Seq Kit (Bioo Scientific, USA) and were then used for paired-end Illumina sequencing (2 × 150 bp) on the Illumina NovaSeq platform. For PacBio sequencing, genomic DNA was fragmented into ~10 kb fragments using G-tubes (Covaris, MA), purified, end-repaired, and ligated with SMRTbell sequencing adapters according to the PacBio protocol (Pacific Biosciences, CA), resulting in a library that was then sequenced on the PacBio RS II platform using standard methods.

Unless otherwise noted, all software was used with default parameters. A total of 8,163,428 Illumina raw reads were generated. The raw reads were quality-trimmed using Trimmomatic v0.36 and assessed using FastQC v0.12.1 (3, 4), yielding 7,690,188 clean reads (*Q*_30_ = 94.50%). A total of 74,601 PacBio reads (*N*_50_ = 11,392 bp) were generated without additional quality control. The PacBio reads were assembled using Unicycler v0.4.8 (5), polished via Pilon v1.24 (6) with Illumina reads, resulting in a singular, closed contig. The genome completeness was assessed using BUSCO v5.4.5 (bacteria_odb10) (7), yielding a completeness of 99.2%. Annotation was conducted using the NCBI Prokaryotic Genome Annotation Pipeline v6.10 (8).

The genome was analyzed with GTDB-Tk v2.3.0 (9) to determine its taxonomic affiliation by calculating average nucleotide identity (ANI) against the GTDB database. The results showed the highest similarity to *Leclercia adecarboxylata* R25 (GCF_006874705.1), with an ANI value of 98.77%, which validated the strain TB492 as *L. adecarboxylata*.

The complete genome of *L. adecarboxylata* TB492 consists of a single circular chromosome of 4,734,680 bp with a G+C content of 56.5%. Annotations identified a total of 4,493 genes, with 84 tRNAs and 25 rRNAs. The general genomic characteristics of TB492 are shown in Table 1.

**TABLE 1:** Genome features of *Leclercia adecarboxylata* strain TB492

Parameter	Result
Genome size (bp)	4,734,680
Completeness (%)	99.2
Genes (total)	4,493
CDSs (coding DNA sequences, total)	4,377
G+C (%)	56.5
Chromosome no.	1
Plasmid no.	0
tRNA no.	84
rRNA no.	9, 8, 8 (5S, 16S, 23S)
ncRNA no.	7

## Data Availability

The raw reads were deposited at the Sequence Read Archive with accession numbers SRR32887236 (Illumina) and SRR35009957 (PacBio) under BioProject number PRJNA1242545. The Whole Genome Shotgun project has been deposited at DDBJ/ENA/GenBank under accession number JBNGCD000000000.2.
